# Primary Hyperparathyroidism in the Pediatric Population: Surgical Considerations and Outcomes: A Narrative Review

**DOI:** 10.3390/diagnostics16040569

**Published:** 2026-02-13

**Authors:** Matija Buzejic, Milan Jovanovic, Vera Zdravkovic, Nikola Slijepcevic, Katarina Tausanovic, Branislav Rovcanin, Sara Ivanis, Vladan Zivaljevic

**Affiliations:** 1 Clinic for Endorcine Surgery, University Clinical Center of Serbia, 11000 Belgrade, Serbia; matijabuzejic@gmail.com (M.B.); milanjovanovicceh@gmail.com (M.J.); saraivaniss@gmail.com (S.I.); 2 University Children’s Hospital Belgrade, 11000 Belgrade, Serbia; 3 School of Medicine, University of Belgrade, 11000 Belgrade, Serbia

**Keywords:** primary hyperparathyroidism, pediatric population, sporadic PHPT, MEN syndrome, parathyroidectomy

## Abstract

Primary hyperparathyroidism (PHPT) in the pediatric population is a rare but clinically important endocrine disorder that poses significant diagnostic and therapeutic challenges. In contrast to adult PHPT, which is often detected incidentally, pediatric patients are frequently symptomatic at diagnosis, with manifestations reflecting prolonged exposure to hypercalcemia and elevated parathyroid hormone levels. Neonatal forms, particularly neonatal severe hyperparathyroidism, represent life-threatening conditions requiring prompt biochemical recognition and urgent intervention. The heterogeneity of clinical presentation and the rarity of the disease contribute to delayed diagnosis and increased risk of end-organ complications. Although hereditary syndromes are proportionally more frequent in children than in adults, sporadic PHPT remains the most common etiology in pediatric patients and is typically caused by a single parathyroid adenoma. Genetically determined forms, including multiple endocrine neoplasia syndromes, hyperparathyroidism–jaw tumor syndrome, and calcium-sensing receptor-related disorders, are often associated with multiglandular disease, earlier onset, and a higher risk of persistence or recurrence. Biochemical confirmation remains the cornerstone of PHPT diagnosis, while diagnostic imaging plays an important role in preoperative localization and surgical planning. High-resolution cervical ultrasound is the preferred first-line imaging modality in pediatric patients due to its excellent diagnostic performance and absence of ionizing radiation. Functional and advanced cross-sectional imaging techniques should be applied in a stepwise manner in selected cases with inconclusive first-line imaging or suspected ectopic disease, balancing diagnostic yield against radiation exposure. Surgical management remains the definitive treatment for pediatric PHPT. The extent of surgery is determined by disease etiology, localization findings, and intraoperative assessment, with focused parathyroidectomy favored in sporadic single-gland disease and more extensive approaches required in genetically determined forms. This review highlights a structured diagnostic and therapeutic pathway for pediatric PHPT, emphasizing the integration of biochemical testing, imaging strategies, genetic evaluation, and individualized surgical management to optimize outcomes and reduce long-term morbidity.

## 1. Introduction

Primary hyperparathyroidism (PHPT) is an endocrine disorder characterized by the overproduction of parathyroid hormone (PTH) by hyperfunctioning parathyroid glands, leading to elevated serum calcium levels [1]. Parathyroid hormone secretion is regulated by serum ionized calcium acting on the calcium-sensing receptor (CaSR) expressed on parathyroid chief cells. Hyperparathyroidism is classically classified into three major subtypes based on pathophysiology: primary, secondary, and tertiary hyperparathyroidism. While these definitions are well established, the exact prevalence of PHPT in the pediatric and adolescent population remains poorly defined.

Primary hyperparathyroidism is considered rare in children, with an estimated incidence of 0.5–5 cases per 100,000 person-years, compared with 50–100 cases per 100,000 person-years in adults. A genetic background is identified in approximately 10–30% of juvenile cases [1,2].

For decades, there has been debate as to whether PHPT in children represents a distinct clinical entity. Although disease manifestations are generally similar to those observed in adolescents and adults, pediatric PHPT often presents with more severe disease and is more frequently accompanied by symptomatic hypercalcemia that may lead to end-organ damage. Several studies have reported that up to 75% of children are symptomatic at the time of diagnosis [3,4,5,6,7]. Whether this reflects true biological differences or diagnostic delay due to disease rarity remains unclear. Patients diagnosed at a younger age are more likely to harbor an underlying hereditary syndrome. Regardless of etiology, surgery remains the treatment of choice in children and young adults, aiming to alleviate symptoms and prevent the development or progression of end-organ damage [8,9]. Other therapeutic strategies, including calcimimetic drugs, may be considered in selected cases, although the optimal duration of treatment and long-term outcomes in the pediatric population remain insufficiently defined [10].

One of the major unresolved challenges in pediatric PHPT relates to diagnostic imaging. Unlike adults, children require a more cautious approach due to increased sensitivity to ionizing radiation, which directly influences the choice and sequencing of localization modalities. Similarly, key clinical questions—such as optimal timing of diagnosis and surgery, appropriate use of medical therapy, selection of imaging techniques, and extent of surgical intervention—remain insufficiently addressed by prospective pediatric studies. Given the rarity of PHPT in children, high-quality randomized trials are difficult to conduct, and current clinical practice is largely based on retrospective data and extrapolation from adult populations.

The originality of this review lies in its integrated, pediatric-specific approach to primary hyperparathyroidism, combining current evidence on genetic background, biochemical diagnosis, imaging strategies, and surgical decision-making into a structured and practical framework. By emphasizing differences between pediatric and adult PHPT and highlighting areas in which adult-based algorithms may be misleading, this review aims to provide clinicians with a focused and clinically applicable guide to the management of PHPT in children and adolescents.

## 2. Methods

A literature search was conducted in PubMed, Scopus, and Web of Science to identify relevant publications related to primary hyperparathyroidism in the pediatric population, published up to December 2025. The search strategy included combinations of keywords such as “primary hyperparathyroidism in children”, “pediatric primary hyperparathyroidism”, and “parathyroidectomy in children.”

This narrative review focused on studies addressing the clinical presentation, diagnostic approach, imaging modalities, genetic background, and therapeutic management of pediatric primary hyperparathyroidism. Priority was given to original research articles, recent review papers, and studies with clear clinical or biological relevance.

Studies were included if they specifically addressed primary hyperparathyroidism in children or adolescents and provided clinically relevant data on diagnosis, treatment strategies, surgical management, or outcomes. Articles not published in English, conference abstracts, and publications with limited relevance to the pediatric population were excluded. The selected literature was qualitatively analyzed and synthesized to provide an integrated overview of current evidence and clinical practice.

## 3. Etiology of Pediatric Primary Hyperparathyroidism

An increasing number of genetic defects have been associated with primary hyperparathyroidism (PHPT). In many cases, germline or somatic mutations in the same gene may lead to the development of multiglandular disease or single parathyroid adenomas.

### 3.1. Sporadic Primary Hyperparathyroidism

Available studies indicate that sporadic PHPT represents the most common etiology in children and adolescents, despite the relatively higher prevalence of genetic forms in this age group compared with adults. Across larger pediatric case series and reviews, sporadic disease accounts for approximately 70–85% of all pediatric PHPT cases, with the remaining proportion attributable to hereditary syndromes such as MEN1, CDC73-related hyperparathyroidism–jaw tumor syndrome, CASR-related disorders, and other rarer genetic entities, which are discussed further in the text [11,12,13,14]. Importantly, even among patients initially classified as having sporadic disease, subsequent genetic testing may identify an underlying germline mutation in a minority of cases [14]. Sporadic PHPT is most frequently associated with single-gland disease, typically due to a solitary parathyroid adenoma, whereas multiglandular involvement is considerably more common in genetically determined forms [15,16].

In terms of age distribution, sporadic PHPT is most commonly diagnosed during adolescence, with a clear peak in the second decade of life, usually between 13 and 18 years of age [17]. This contrasts with neonatal and early childhood presentations, which are more suggestive of a genetic etiology. The delayed diagnosis often observed in sporadic pediatric PHPT reflects the nonspecific and heterogeneous nature of its clinical manifestations. Unlike adults, in whom PHPT is frequently identified incidentally, most affected children are symptomatic at the time of diagnosis [18].

### 3.2. Multiple Endocrine Neoplasia Type 1 (MEN1)

Primary hyperparathyroidism is the most frequent and usually the earliest manifestation of multiple endocrine neoplasia type 1 (MEN1). MEN1 is an autosomal dominant disorder caused by pathogenic variants in the MEN1 gene, and is associated with an increased risk of developing PHPT, pituitary neuroendocrine tumors (PitNETs), and duodenopancreatic neuroendocrine neoplasms; also, it may present with dermatologic manifestations such as angiofibromas or lipomas [19,20].

PHPT may be diagnosed at a very early age, as young as 4 years, and surgical intervention during childhood has historically occurred in 31% to 55% of patients [21,22]. However, symptomatic PHPT is rare prior to the third decade of life [23]. Surveillance should begin at the age of 10 years, with annual measurement of serum calcium levels.

The optimal timing and surgical approach in young patients with MEN1 remain controversial. Even after successful initial surgery, the risk of recurrent PHPT at 10 years is approximately 41%, and nearly half of patients experience recurrence within 15 years [19,24]. Consequently, repeat surgery is frequently required during an affected individual’s lifetime despite adequate initial intervention, and the benefits of an early aggressive approach must be carefully balanced against its risks, including permanent hypoparathyroidism. Adults with PHPT are typically offered subtotal (3.5-gland) parathyroidectomy with transcervical thymectomy. In younger patients, unilateral (two-gland) exploration may be considered. According to the recommendations of the German Association of Endocrine Surgeons (CAEK), subtotal or total parathyroidectomy with thymectomy and autotransplantation is indicated in MEN1 patients. To reduce the risk of permanent hypoparathyroidism, selective resection of enlarged parathyroid glands may be performed; however, patients must be informed about the increased risk of reoperation for recurrent PHPT [25].

### 3.3. Multiple Endocrine Neoplasia Type 2A (MEN2A)

Gain-of-function pathogenic variants in the RET gene increase the likelihood of developing three endocrine tumors pathognomonic for MEN2: medullary thyroid carcinoma (MTC), pheochromocytoma (PCC), and, in MEN2A, primary hyperparathyroidism. The American Thyroid Association guidelines further stratify MEN2 patients into subcategories based on the specific RET mutation present and the associated severity and malignancy risk. According to these guidelines, annual serum calcium measurement is recommended from the age of 11 years in patients with RET codon 918, 634, and 883 mutations, and from the age of 16 years in individuals with all other exon mutations [26].

Surgical treatment is the definitive curative option for these patients; however, the timing of parathyroid surgery often does not coincide with thyroid surgery performed for medullary thyroid carcinoma.

It should also be noted that other genetic mutations and well-defined syndromes, such as MEN4 and MEN5, have been described. MEN4 is associated with germline pathogenic variants in the cyclin-dependent kinase inhibitor 1B (CDKN1B) gene located on chromosome 12p13.1 [27]. Patients with MEN4 exhibit a tumor spectrum similar to that observed in MEN1, including primary hyperparathyroidism (PHPT) in approximately 75–80% of cases [28]. Importantly, MEN4 manifestations tend to occur at a later mean age compared with MEN1, with PHPT typically presenting between 45 and 56 years of age [29].

MEN5 is inherited in an autosomal dominant variant, and its most common manifestation is adrenomedullary pheochromocytoma, which is frequently bilateral and/or multicentric. Germline pathogenic variants in the MAX gene have been identified in MEN5, and PHPT may also occur in affected individuals. Due to the limited available data, annual measurement of serum calcium levels in patients older than 10 years appears to represent a sufficient surveillance strategy [30].

### 3.4. Hyperparathyroidism–Jaw Tumor (HPT-JT) Syndrome

The CDC73 gene (formerly HRPT2) encodes parafibromin, a tumor suppressor protein. Pathogenic variants in the CDC73 gene cause HPT-JT syndrome, a condition characterized by parathyroid tumors, most commonly presenting as a single parathyroid adenoma; however, in 10–15% of cases, parathyroid carcinoma may be the underlying cause of PHPT [31]. Variants in the CDC73 gene are identified in up to 70% of individuals with parathyroid carcinoma. Approximately one-third of these individuals inherit the variant from a parent as a germline mutation, whereas in the remaining two-thirds, the variant arises during the individual’s lifetime as a somatic mutation [32,33].

In addition to PHPT, patients with HPT-JT syndrome frequently develop ossifying fibromas of the mandible or maxilla, which occur in approximately 30–40% of affected individuals [34]. Annual monitoring of serum calcium levels should begin at the age of 5 years.

### 3.5. Neonatal Severe Hyperparathyroidism and Familial Hypocalciuric Hypercalcemia

Neonatal severe hyperparathyroidism (NSHPT) occurs as a result of inactivating mutations in the gene encoding the calcium-sensing receptor (CASR). Reduced sensitivity of the calcium-sensing receptor to extracellular calcium leads to excessive parathyroid hormone secretion and, consequently, severe hypercalcemia. While NSHPT is caused by homozygous or compound heterozygous mutations, heterozygous mutations in the same gene result in familial hypocalciuric hypercalcemia (FHH) [35].

Patients with NSHPT usually present within the first two weeks of life with serum calcium levels exceeding 4.5 mmol/L, and parathyroid hormone levels are elevated in more than 80% of affected individuals [36]. If diagnosis and treatment are delayed, NSHPT may be associated with high mortality or irreversible neurodevelopmental, renal, skeletal, or cardiovascular complications [37]. Neurological sequelae, including mild intellectual disability, microcephaly, and epilepsy, have been reported even 10 years after parathyroidectomy, despite surgery being performed on the 11th postnatal day [38].

Familial hypocalciuric hypercalcemia is rarely detected in infancy, and the majority of affected individuals are asymptomatic (approximately 70%). It is inherited in an autosomal dominant manner, and three distinct genetic disorders causing FHH have been identified:

(a) FHH1, caused by loss-of-function mutations in the CASR gene, accounting for approximately 65% of all FHH cases;
(b) FHH2, caused by loss-of-function mutations in GNA11, encoding the α11 subunit of the G protein, accounting for fewer than 5% of cases;
(c) FHH3, caused by mutations in the AP2S1 gene, which encodes the adaptor-related protein complex 2 sigma-1 subunit, accounting for approximately 20% of FHH patients in whom CASR mutations are not detected [39,40].

Table 1 summarizes the causes of PHPT in children, which are discussed individually below, together with therapeutic considerations for each patient group.

## 4. Diagnostic Challenge

Pediatric PHPT is often associated with a more severe clinical course than PHPT in adults. This is usually related to its heterogeneous presentation, the broad spectrum of symptoms that children may not be able to clearly describe, and the rarity of the condition, which together contribute to delayed clinical recognition and diagnosis.

### 4.1. Clinical Manifestation

In neonatal forms of primary hyperparathyroidism (PHPT), such as NSHPT, affected individuals usually present within the first two weeks postpartum with markedly elevated serum calcium levels [36,37,38]. The clinical presentation in the neonatal period is often dramatic and life-threatening. Common features include failure to thrive, persistent constipation, lethargy or increased irritability, respiratory distress syndrome due to hypotonia of the respiratory muscles, and profound skeletal demineralization with bone deformities, fractures, and generalized hypotonia. Severe hypercalcemia in this age group may also lead to dehydration, polyuria, feeding difficulties, and impaired neuromuscular function.

In contrast, older children and adolescents more frequently present with heterozygous genetic alterations or sporadic disease, which are typically associated with more moderate biochemical abnormalities and a broader spectrum of musculoskeletal and renal manifestations. While higher calcium and PTH levels generally correlate with disease severity, overlap between clinical phenotypes exists, particularly in genetically determined forms. Disease severity is influenced by the duration of exposure to elevated calcium and parathyroid hormone levels before diagnosis is established. Musculoskeletal involvement represents one of the most common and prominent clinical manifestations. Some authors have reported musculoskeletal pain—typically diffuse, nonspecific, and dull aching in nature—in up to 90% of pediatric patients at presentation [41]. Additional skeletal manifestations include genu valgum, bone cysts, reduced bone mineral density, osteitis fibrosa cystica, and brown tumors, reflecting high bone turnover during growth. In advanced or untreated cases, pathological fractures and impaired linear growth may occur, particularly during periods of rapid skeletal development such as puberty [42].

Renal and gastrointestinal manifestations are also frequently encountered in pediatric PHPT. Hypercalcemia may impair renal concentrating ability and promote calcium stone formation, leading to nephrolithiasis, nephrocalcinosis, hematuria, polyuria, and flank or abdominal pain [43]. In some pediatric series, nephrolithiasis has been identified in up to 50% of affected patients, underscoring the burden of renal complications in this population [44]. Gastrointestinal symptoms are variable and may include recurrent abdominal pain, nausea, vomiting, anorexia, constipation, and weight loss. Although rare, acute abdomen secondary to hypercalcemia-induced acute pancreatitis has been reported as an initial manifestation of PHPT in children and adolescents and should be considered in cases of unexplained abdominal pain accompanied by hypercalcemia [45]. Neuropsychiatric symptoms such as fatigue, irritability, impaired concentration, mood changes, and reduced school performance have also been described, particularly in adolescents, further complicating timely diagnosis.

When any of the above manifestations occur—either in isolation or in combination—measurement of serum calcium and parathyroid hormone (PTH) levels is recommended. In children with a known family history of hyperparathyroidism or associated genetic syndromes, biochemical screening with serum calcium and PTH assessment should be performed proactively, even in the absence of overt clinical symptoms.

Age-adjusted reference ranges for serum calcium concentrations are summarized in Table 2 [46].

### 4.2. Preoperative Imaging

Diagnostic imaging plays a relevant role in the management of pediatric PHPT, primarily for preoperative localization rather than for establishing the diagnosis itself, which remains biochemical. When PHPT is caused by a single parathyroid adenoma, high-resolution cervical ultrasound (US) is widely accepted as the first-line imaging modality in pediatric patients. It is non-invasive, free of ionizing radiation, readily available, and highly operator-dependent. Recent pediatric series have demonstrated excellent performance of ultrasound, with reported sensitivities approaching 100% for single-gland disease when performed by experienced operators [47]. Ultrasound is particularly effective for identifying eutopic parathyroid adenomas located posterior or inferior to the thyroid gland, and it also provides valuable information about concomitant thyroid pathology, which is not uncommon in adolescents with PHPT [19].

Technetium-99m sestamibi (99mTc-MIBI) scintigraphy, often combined with SPECT or SPECT/CT, has traditionally been considered a complementary first-line modality in adults; however, its role in children is more controversial. While MIBI scintigraphy can provide functional information and improve specificity in selected cases, several pediatric studies have shown that it adds little diagnostic value when ultrasound findings are clearly positive [48,49]. Importantly, children are more sensitive to ionizing radiation, and minimizing cumulative radiation exposure is a key principle in pediatric imaging. Evidence suggests that routine use of MIBI scintigraphy may be unnecessary in pediatric patients with concordant biochemical findings and a clearly localized lesion on ultrasound [50]. Its use may be reserved for cases with negative or equivocal ultrasound results, suspected multiglandular disease, prior failed surgery, or recurrent hyperparathyroidism. In these contexts, MIBI imaging can help refine surgical planning rather than increase sensitivity per se [49,50,51].

More recently, 18F-fluorocholine (FCH) positron emission tomography (PET) combined with computed tomography (CT) has been shown to be more accurate than ultrasonography or 99mTc-sestamibi single-photon emission computed tomography (SPECT) in cases of recurrent disease or non-localized parathyroid lesions [52].

Advanced cross-sectional imaging techniques, such as four-dimensional computed tomography (4D-CT) and magnetic resonance imaging (MRI), are increasingly used as second- or third-line modalities in complex pediatric cases (non US detectable, recurrent disease) [53]. Four-dimensional CT offers high spatial resolution and excellent sensitivity for small or ectopic parathyroid adenomas, particularly in mediastinal or intrathymic locations; however, its use is limited by relatively high radiation doses and should be restricted to selected cases in which first-line imaging fails. MRI, including dynamic contrast-enhanced protocols, represents a radiation-free alternative with moderate sensitivity and is especially useful in young children or in patients requiring repeated imaging [54].

**Figure 1:**
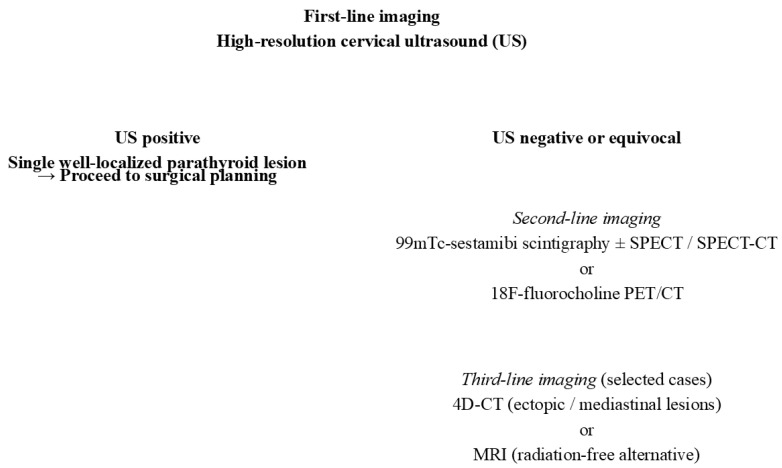
Imaging modalities of PHPT presented as figure.

In pediatric patients with persistent or recurrent PHPT, progression to advanced imaging is guided by specific clinical and imaging criteria, including negative or equivocal ultrasound findings, suspected multiglandular disease, prior unsuccessful surgery, or suspicion of ectopic parathyroid tissue. In this setting, 18F-fluorocholine PET/CT is increasingly favored due to its high sensitivity at relatively lower radiation exposure compared with 4D-CT, particularly when precise localization is required after failed first-line imaging. Four-dimensional CT is generally reserved for selected cases in which PET imaging is unavailable or inconclusive, or when detailed anatomical resolution is necessary for surgical planning, especially in mediastinal or ectopic locations. The stepwise algorithm of localization methods is illustrated in Figure 1.

## 5. Management of Pediatric Primary Hyperparathyroidism

Surgery represents the definitive treatment for well-diagnosed primary hyperparathyroidism, particularly in patients with precise preoperative imaging localization, and across all age groups. The type and extent of surgical resection are determined on an individual basis. In cases of single-gland disease, focused parathyroidectomy is the gold standard; however, in children with genetically determined forms of PHPT, a more extensive surgical approach is required and must be tailored to the specific underlying disorder.

Genetic testing should be considered early in the diagnostic workup of pediatric PHPT, even in patients without an apparent family history, as hereditary forms are not uncommon in this age group. Testing is particularly relevant in younger patients, those with multiglandular disease, recurrent or persistent hyperparathyroidism, or markedly elevated calcium or PTH levels. Identification of pathogenic variants, such as MEN1 or CASR mutations, has important implications for surgical planning, as it may influence the extent of initial surgery or, in the case of CASR-related disorders, help avoid unnecessary parathyroidectomy. Incorporating genetic testing before definitive surgical intervention supports individualized management and may reduce the risk of persistence or recurrence.

### 5.1. Surgery—Parathyroidectomy

In children with sporadic PHPT, once biochemical confirmation and accurate preoperative localization have been achieved, parathyroidectomy represents the treatment of choice. Surgical management may be performed either as minimally invasive parathyroidectomy or as conventional open surgery. Historically, bilateral neck exploration with identification of all four parathyroid glands was routinely performed, even in cases suspected to involve single-gland disease [55,56]. However, with advances in preoperative localization techniques and the widespread adoption of intraoperative parathyroid hormone (ioPTH) monitoring, this approach has become increasingly less common [57]. Systematic use of intraoperative PTH monitoring facilitates a focused and tissue-sparing surgical approach, allowing preservation of uninvolved cervical compartments in patients with sporadic PHPT and potentially reducing morbidity in the event of reoperation.

A significant intraoperative decline in PTH levels following excision of the targeted gland is considered indicative of surgical success, allowing the procedure to be completed as a focused parathyroidectomy without the need for extensive cervical exploration [57,58]. In adults, a significant intraoperative PTH decline is commonly defined as a reduction of at least 50% from the baseline level; however, no universally accepted cutoff has been established for the pediatric population [59,60]. A decline of ≥50% in intraoperative PTH levels should be interpreted with caution in pediatric patients and may not be sufficient as a standalone indicator of surgical cure, particularly given the higher prevalence of multiglandular and genetically determined disease and the associated risk of persistence or recurrence. Available pediatric data are limited to small retrospective series, and consequently, several pediatric studies have adopted additional or more stringent criteria, such as obtaining a 20 min intraoperative PTH sample and requiring a greater percentage decline. These approaches are largely extrapolated from adult criteria but appear to provide reliable intraoperative guidance in pediatric cohorts. This more conservative strategy reflects the relatively higher frequency of multiglandular or ectopic parathyroid disease in children, in whom less stringent criteria may be misleading [61]. Further prospective studies are needed to define pediatric-specific intraoperative PTH thresholds.

This surgical strategy, however, is not applicable to genetically determined forms of PHPT, particularly in patients with multiple endocrine neoplasia type 1 (MEN1) and multiple endocrine neoplasia type 2A (MEN2A). In these conditions, with multiglandular involvement, targeted parathyroidectomy is associated with a high risk of persistent or recurrent disease. Consequently, a more extensive surgical approach is recommended. According to the guidelines of the German Association of Endocrine Surgeons (CAEK), the procedure of choice in patients with MEN1-associated PHPT is subtotal or total parathyroidectomy, combined with cervical thymectomy and autotransplantation of parathyroid tissue. This strategy aims to reduce the risk of recurrence while preserving long-term calcium homeostasis, although it requires careful postoperative monitoring for hypocalcemia and long-term follow-up [26].

Furthermore, in patients with MEN1 and MEN2A syndromes, intraoperative PTH monitoring should be interpreted with caution. An early decline in PTH levels—even if exceeding 50%—does not reliably indicate complete removal of all hyperfunctioning tissue. Therefore, reliance on strict intraoperative PTH criteria alone is insufficient in these patients, and a planned extensive surgical approach (subtotal or total parathyroidectomy, with or without autotransplantation) remains the recommended strategy, regardless of intraoperative PTH dynamics [62,63].

In HPT-JT syndrome, parathyroid disease is often aggressive and carries an increased risk of recurrence and parathyroid carcinoma. Consequently, minimally invasive parathyroidectomy is generally discouraged, even in apparently single-gland disease, and a more cautious surgical approach with en bloc resection of the affected gland and careful cervical exploration is recommended [64]. Neonatal severe hyperparathyroidism, requires definitive treatment, such as total parathyroidectomy, which is often performed early in the neonatal period; autotransplantation is generally avoided because of the risk of persistent or recurrent hyperparathyroidism. Medical management may provide temporary stabilization as a bridge to surgery in these cases [65,66]. In contrast, familial hypocalciuric hypercalcemia is a benign condition associated with lifelong mild hypercalcemia and relative hypocalciuria, in which parathyroid hormone levels are typically normal or only mildly elevated. In these patients, parathyroidectomy is not indicated, as surgical intervention does not normalize serum calcium levels and should be avoided; management consists of conservative follow-up and confirmation of the diagnosis by biochemical and genetic testing [67].

### 5.2. Pharmacological Treatment

Medical therapy may be used as a temporary measure or as a bridge to surgery, particularly to control hypercalcemia and stabilize serum calcium levels before definitive intervention. Such measures include adequate hydration, loop diuretics (e.g., furosemide), and bisphosphonates, which reduce bone resorption and transiently lower serum calcium concentrations [68,69,70]. These approaches are considered supportive and temporary, as they do not address the underlying cause of parathyroid hormone excess.

In contrast, calcimimetics, particularly cinacalcet, have emerged as a potential therapeutic option in selected patients with genetically determined forms of PHPT, most notably those with multiple endocrine neoplasia type 1 (MEN1) [71]. Calcimimetics act by increasing the sensitivity of the calcium-sensing receptor on parathyroid cells, thereby suppressing PTH secretion and reducing serum calcium levels. Their role is primarily reserved for patients who are poor surgical candidates, refuse surgery, or have persistent or recurrent hyperparathyroidism after surgical treatment [72]. Although calcimimetics have demonstrated efficacy in reducing serum calcium levels in adult patients with PHPT, evidence supporting their long-term use in pediatric populations remains limited. Based on available evidence, the use of calcimimetics in pediatric population should be approached with caution especially in infants with persistent and severe primary. In such cases, treatment may be considered only when conventional measures have failed and surgery is not feasible, and it requires informed discussion with the family. During therapy, serum and ionized calcium levels are required, especially during dose titration, given the risk of hypocalcemia. In our interpretation of published literature, gastrointestinal adverse effects such as nausea, vomiting, and diarrhea are the most frequently reported and require careful clinical surveillance. Younger age appears to be associated with a higher incidence of adverse events, underscoring the need for individualized dosing and intensified monitoring during calcimimetic therapy [71,72,73].

## 6. Discussion

Primary hyperparathyroidism (PHPT) in the pediatric population represents a rare but clinically significant endocrine disorder characterized by heterogeneous presentation, frequent diagnostic delay, and a high burden of disease-related complications at the time of diagnosis. Unlike adult PHPT, which is often detected incidentally, pediatric PHPT is predominantly symptomatic and commonly associated with skeletal, renal, gastrointestinal, and neuropsychiatric manifestations. Disease severity is closely related to the duration of exposure to hypercalcemia and elevated parathyroid hormone levels, underscoring the importance of early recognition and timely intervention. Neonatal forms, particularly neonatal severe hyperparathyroidism, represent life-threatening conditions requiring urgent diagnosis and definitive treatment [1,2,3,4,5,6,7,8,9,10,11,36].

Although genetic forms of PHPT are relatively more frequent in children than in adults, sporadic disease remains the most common etiology in pediatric patients and is typically caused by a single parathyroid adenoma. In contrast, genetically determined forms—such as MEN1, MEN2A, HPT-JT syndrome, and CASR-related disorders—are frequently associated with multiglandular disease, earlier onset, and a higher risk of recurrence, necessitating a distinct diagnostic and therapeutic approach. Accurate etiological classification, supported by genetic testing when appropriate, is therefore important for guiding surgical strategy, long-term surveillance, and family counseling [20,21,22,23,24,25,26,27,28,29,30,31,32,33,34,35,36,37,38,39,40,41].

Diagnostic imaging plays significant role in the management of pediatric PHPT by enabling precise preoperative localization rather than establishing the diagnosis itself, which remains biochemical. High-resolution cervical ultrasound is the preferred first-line imaging modality due to its excellent performance, lack of ionizing radiation, and ability to assess concomitant thyroid pathology. Functional imaging modalities and advanced cross-sectional techniques should be reserved for selected cases, particularly when first-line imaging is inconclusive, disease is recurrent, or ectopic parathyroid tissue is suspected. A stepwise imaging algorithm is essential to minimize radiation exposure while maximizing diagnostic accuracy in this vulnerable population [47,48,49,50,51,52,53,54].

Surgery remains the central component of definitive treatment for pediatric PHPT. In sporadic single-gland disease, focused parathyroidectomy guided by accurate preoperative localization and intraoperative PTH monitoring is associated with excellent outcomes. However, strict reliance on intraoperative PTH criteria must be approached with caution in children, particularly in those with suspected multiglandular or genetically determined disease. In MEN1 and MEN2A, limited parathyroidectomy is associated with a high risk of persistence or recurrence, and planned more extensive surgical strategies are recommended. Similarly, in HPT-JT syndrome, the aggressive nature of parathyroid disease and the risk of carcinoma necessitate an oncologically cautious surgical approach. In contrast, familial hypocalciuric hypercalcemia should be recognized as a benign condition in which parathyroidectomy is contraindicated [55,56,57,58,59,60,61,62,63,64,65,66,67].

After parathyroidectomy, careful and structured follow-up is important to ensure early detection and management of postoperative complications, particularly hypocalcemia and hungry bone syndrome. Intensive monitoring of total and ionized serum calcium is recommended during the first 24–72 h after surgery, initially every 4–6 h and subsequently once or twice daily until biochemical stability is achieved [74,75,76]. Concurrent assessment of serum phosphate, magnesium, and parathyroid hormone levels is important, as hypomagnesemia and an abrupt decline in PTH may exacerbate and prolong postoperative hypocalcemia. Patients with high preoperative PTH levels, elevated alkaline phosphatase, or radiological evidence of skeletal involvement are at increased risk of developing hungry bone syndrome and may benefit from early prophylactic administration of oral calcium and active vitamin D metabolites. Following hospital discharge, continued outpatient surveillance is required to confirm sustained biochemical remission and to allow early identification of persistent or recurrent disease, particularly in children with hereditary forms of primary hyperparathyroidism [76].

Medical therapy plays a supportive role in pediatric PHPT and may be used as a temporary measure to stabilize hypercalcemia or as an alternative in selected patients who are not candidates for surgery. While conventional measures provide only transient biochemical control, calcimimetics have emerged as a valuable option in selected genetically determined cases, particularly MEN1-associated PHPT. Nevertheless, surgical intervention remains the only curative treatment for the majority of pediatric patients [68,69,70,71,72].

## 7. Conclusions

**Figure 2:**
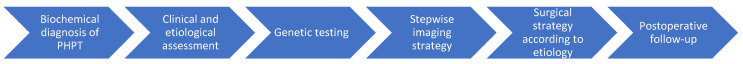
Outlines a structured clinical pathway beginning with biochemical confirmation of primary hyperparathyroidism, followed by clinical and etiological assessment to identify potential hereditary forms. Genetic testing is selectively incorporated to guide further management. Preoperative localization is performed using a stepwise imaging strategy that prioritizes ultrasound and limits radiation exposure. Surgical strategy is tailored according to the underlying etiology and disease extent, and postoperative follow-up focuses on early detection of complications and long-term disease surveillance. Familial hypocalciuric hypercalcemia should be systematically excluded early in the diagnostic algorithm (Figure 2), as parathyroidectomy is contraindicated in this condition.

In summary, optimal management of pediatric PHPT requires a multidisciplinary, individualized approach that integrates careful clinical assessment, biochemical confirmation, judicious use of imaging, tailored surgical strategy, and long-term follow-up. Improved awareness of this rare condition, together with advances in genetic diagnostics and surgical techniques, may further enhance outcomes and reduce disease-related morbidity in affected children and adolescents. All steps of diagnosis and treatment are presented in Figure 2.

## Figures and Tables

**Table 1:** Summary of Causes, Clinical Features, and Onset of PHPT in Children.

Syndrome/Condition	Responsible Gene	Type of Mutation	Typical Manifestation	Age of Onset	Recommendation for Calcium and PTH Monitoring
Sporadic PHPT	None identified	Somatic (adenoma-associated)	Single-gland parathyroid adenoma; symptomatic hypercalcemia	Adolescence (peak in second decade)	Biochemical testing at diagnosis; postoperative monitoring according to clinical course
MEN1	MEN1	Germline, loss-of-function	Multiglandular PHPT, PitNETs, pancreatic NETs	Childhood; symptomatic disease usually after 20–30 years	Annual serum calcium from age 10 years
MEN2A	RET	Germline, gain-of-function	PHPT, MTC, pheochromocytoma	Adolescence or adulthood	Annual serum calcium from age 11 years (RET 918, 634, 883); from age 16 years for other mutations
MEN4	CDKN1B	Germline, loss-of-function	MEN1-like spectrum, PHPT common	Typically adulthood (45–56 years)	Surveillance in adulthood; no pediatric routine screening
MEN5	MAX	Germline pathogenic variants	Pheochromocytoma, occasional PHPT	Variable, usually adolescence/adulthood	Annual serum calcium from age 10 years
HPT-JT syndrome	CDC73	Germline or somatic	Single adenoma or parathyroid carcinoma; jaw tumors	Childhood or adolescence	Annual serum calcium from age 5 years
NSHPT	CASR	Homozygous or compound heterozygous	Severe neonatal hypercalcemia	Neonatal period	Immediate and continuous biochemical monitoring
FHH (types 1–3)	CASR, GNA11, AP2S1	Heterozygous loss-of-function	Mild, usually asymptomatic hypercalcemia	Any age	Periodic calcium monitoring; PTH usually not routinely required

PHPT, primary hyperparathyroidism; MEN, multiple endocrine neoplasia; MEN1, multiple endocrine neoplasia type 1; MEN2A, multiple endocrine neoplasia type 2A; MEN4, multiple endocrine neoplasia type 4; MEN5, multiple endocrine neoplasia type 5; HPT-JT, hyperparathyroidism–jaw tumor syndrome; NSHPT, neonatal severe hyperparathyroidism; FHH, familial hypocalciuric hypercalcemia; PitNETs, pituitary neuroendocrine tumors; NETs, neuroendocrine tumors; MTC, medullary thyroid carcinoma; PTH, parathyroid hormone.

**Table 2:** Age-specific reference ranges for total serum calcium.

Age Group/Condition	Serum Calcium Value (Range) mmol/L
Newborns (0–90 days)	2.0–2.8
Infants (91–180 days)	2.2–2.8
Infants (181–365 days)	2.2–2.9
Early childhood (1–3 years)	2.2–2.8
Childhood (4–11 years)	2.2–2.7
Children (>12 years)	2.2–2.8

## Data Availability

No new data were created or analyzed in this study. Data sharing is not applicable to this article.
